# Translating imaging biomarkers into clinical practice

**DOI:** 10.1186/1470-7330-15-S1-O9

**Published:** 2015-10-02

**Authors:** James PB O'Connor

**Affiliations:** 1 Institute of Cancer Sciences, University of Manchester, Manchester, M20 4BX, UK

## 

Biomarkers are ‘*objectively measured and evaluated indicators of normal biological processes, pathogenic processes or pharmacologic responses to a therapeutic intervention*’ that identify increased or decreased risk of patient benefit or harm [1]. Imaging biomarkers (IB) are integral to cancer healthcare and research. In oncology, patient management relies heavily on using ordered categorical IBs to stage patients (e.g. assignation of T, N and M status) and to monitor therapeutic efficacy (e.g. objective response, measured by RECIST 1.1 or equivalent criteria) [2,3]. IBs are also used to measure toxicity in cancer patients. For example, SPECT quantification of cardiac ejection fraction is an important biomarker of drug-induced cardiotoxicity [4].

The role of IBs in oncology continues to increase in the era of personalised medicine. Every year thousands of imaging studies develop IBs and test their role as putative prognostic, predictive, monitoring and radiation planning biomarkers - both for use in healthcare and in clinical trials of novel drug or radiotherapy treatments [5]. Some IBs modify existing metrics. For example, basing response criteria largely on ^18^F-FDG PET signal changes rather than size changes (as in PERCIST v RECIST) may stratify patients differently but still uses the same conceptual biomarker, namely objective response [6].

In distinction, many other IBs derive parameters that measure novel aspects of tumour molecular biology, pathophysiology or structural morphology. These IBs are usually designed to quantify an unmet clinical need, such as the hallmarks of cancer that are targets for drug development. Examples include optical imaging of deoxy-Hb and oxy-Hb ratios as a biomarker of hypoxia; measuring ^13^C-bicarbonate/CO_2_ ratios through dynamic nuclear polarisation to map tumour pH; measuring changes in glucose metabolism through quantifying percentage reduction in ^18^F-FDG PET SUV_max_ ; measuring changes in vascular function through quantifying percentage reduction in *K*^trans^; or measuring tumour heterogeneity by texture, fractal or other feature-based analyses [7-9].

Unfortunately, translation of new IBs has been disappointing. Quantitative IBs in particular have been slow to cross the translational gaps to become useful decision making tools in drug development (pharmacodynamic or PD IB) or in altering healthcare (as companion diagnostics, or for screening, response, monitoring or outcome). The key reason that IBs have failed to make substantial impact is the lack of clear roadmap for IB validation and qualification. IBs have several important differences from the more familiar biospecimen-derived biomarkers and require a different validation roadmap tailored to the strengths and limitations of IB. Recognising this need, *Cancer Research UK* and the *European Organization for Research and Treatment of Cancer* (*EORTC*) have sponsored an international consensus effort to devise a roadmap and produce key recommendations for the design, performance, governance and publication of future IB studies [10].

This talk aims to:

1. Challenge delegates in their understanding of what constitutes an IB

2. Introduce current thinking around how IBs should be validated and qualified (the ‘imaging biomarker roadmap for use in cancer studies’)

3. Provide a range of examples that highlight the successes and failures of many popular and emerging IBs
